# Cortical Gray Matter Volume Associations with Dietary Fiber and Calorie Density: A Pilot Study

**DOI:** 10.21203/rs.3.rs-5338934/v1

**Published:** 2024-11-28

**Authors:** Raghav Pallapothu, Roger D. Newman-Norlund, Makayla Gibson, Pranesh Rajesh Kannan, Chris Rorden, Leo Bonilha, Julius Fridriksson

**Affiliations:** 1 University of South Carolina, Department of Psychology, College of Arts and Sciences, Columbia, SC; 2 University of South Carolina, Department of Communication Sciences, Arnold School of Public Health, Columbia, SC; 3 Emory School of Medicine, Department of Neurology, Atlanta, GA

**Keywords:** brain, gray matter volume, fiber, calorie count

## Abstract

Alzheimer’s disease and related dementia are associated with significant societal costs and economic burdens and have been associated with decreases in cortical gray matter volume. This pilot cross-sectional study investigates the relationship between total fiber concentration in diet, total calorie density per diet, and regional GMV in a cohort of 190 patients aged 20 to 79 years, utilizing data from the 2015 National Health Interview Survey Supplement and T1-weighted magnetic resonance imaging (MRI) collected at the McCausland Center for Brain Imaging. Gray matter volumes were quantified using the CAT12 voxel-based morphometry toolbox and statistical correlations between GMV and dietary measures were assessed using Spearman’s rank order correlation coefficient. Our exploratory analysis revealed a significant positive correlation between dietary fiber consumption and GMV in 16 different areas, including the right Hippocampus (r (190) = 0.196, p = 0.003) and left Hippocampus (r (190) = 0.135, p = 0.032), when controlling for age and race of participants. Our analyses revealed significantly negative correlations between total calorie density per diet and localized GMV in 3 different brain areas, including the right Precentral Gyrus (r (190) = −0.15, p = 0.039) and right Thalamic Pulvinar (r (190) = −0.155, p = 0.033), when controlling for participant race. Together, our initial findings suggest that dietary fiber and calorie density are associated with GMV even after controlling for relevant demographic variables. If replicated, these findings could have important implications for dietary advice given to individuals at risk for developing MCI or more serious forms of dementia.

## Introduction:

Dementia is one of the most common neurological disorders, with over 55 million people worldwide living with this diagnosis in 2020, and over 10 million new cases occurring annually. Even with a widespread awareness and diagnosis of this disorder, it is hypothesized that still, approximately 75% of people living with dementia have not received a diagnosis (World Alzheimer Report 2022: Life after Diagnosis: Navigating Treatment, Care and Support, 2022). Alzheimer’s Disease, the most prevalent form of dementia, is typically characterized by an abnormally large loss of gray matter across the brain, including areas of the hippocampal and temporal regions, as well as regions near the thalamus (van de Mortel et al., 2021). Thus, gray matter, responsible for memory functions, emotional regulation, movement, and muscle control, is seemingly linked as a potential biomarker of neurodegenerative disorders like Alzheimer’s and dementia. As a result of the scope and severity of dementia’s effects on gray matter, there is a demand for suitable treatment and practices that could further decrease the rate of gray matter decay.

Recent literature has suggested that the consumption of Mediterranean diets consisting of high concentrations of Omega-3 fatty acids have been associated with a decrease in the progression of Alzheimer’s, suggesting that gray matter is more likely preserved in those who consume such. Specifically, fatty acids, polyphenols, and flavonoids from plant origin play a critical role, due to their largely anti-inflammatory and antioxidant properties that seemingly improve gut-brain axis health, affecting the progression of neurodegenerative disorders (“Mediterranean Diet: The Role of Long-Chain ω-3 Fatty Acids in Fish; Polyphenols in Fruits, Vegetables, Cereals, Coffee, Tea, Cacao and Wine; Probiotics and Vitamins in Prevention of Stroke, Age-Related Cognitive Decline, and Alzheimer Disease,” 2019). These Omega-3 fatty acids also lead to the release of eicosapentaenoic acid (EPA) and docosahexaenoic acid (DHA), types of short-chain fatty acids (SCFA), which are the main anti-inflammatory components of the fatty acids (Calder, 2010). Recent research also states that foods with lower dietary inflammation indexes (DII) are more beneficial for cognitive health. Participants in the highest DII score tertile (indicating a diet with the highest proinflammatory potential) were three times more likely to develop dementia compared to those in the lowest tertile (95% CI 1.2–7.3; p = 0.014). Similarly, the same investigation of 1,059 individuals found that each unit increase in the DII was associated with a 21% increase in dementia incidence (Diet Inflammatory Index and Dementia Incidence, n.d.). However, recent research investigating the gut microbiota has suggested that dietary fiber secretes short-chain fatty acids like Omega-3 fatty acids. Dietary fiber is the consumable parts of carbohydrates that are typically resistant to digestion in the small intestine, including polysaccharides, oligosaccharides, and other plant substances (Fuller et al., 2016). When dietary fiber is fermented by gut bacteria, it has been reported to lead to the release of SCFAs like acetate and butyrate, which are known to reduce systemic inflammation in the gut, creating a similar reaction to that of Omega-3 fatty acids (Fu et al., 2022).

Due to the decrease in consumption of dietary fiber, the global epidemic of obesity and other metabolic disorders has grown, contributing to dietary inflammation. In patients with obesity, there is a large accumulation of a specific adipose tissue, which releases a variety of pro-inflammatory adipokines like leptin, further increasing the risk of neurodegenerative disorders in patients with low fiber consumption, possibly leading to gray matter volume loss (Lee et al., 2013). Furthermore, evidence suggests that dietary fiber could modulate metabolic risk factors, with new literature suggesting that dietary fiber impacts insulin resistance and the sympathetic nervous system: the main psychophysiological pathways involved in cardiovascular risk. Longitudinal studies further show that fiber intake of 10 grams/1000 kcal/day or 20 grams/day in several trials has been associated with body weight loss of 1.2- 3.6 kilograms after 8-12 years from the onset of fiber intake. In these studies, regardless of baseline body weight classification (morbid obesity, obesity, normal weight, etc.), fiber intake was associated with beneficial results in weight management, BMI, body fat percentage, and waist circumference (Bozzetto et al., 2018).

The impact of dietary fiber is not limited to purely metabolic disorders, however. Recent findings suggest that increased intake of Natural Dietary Fiber (NDF) significantly alters the intestinal environment, influencing the gastrointestinal immune and endocrine system responses. These drastic gut changes are linked to the alteration of the physiology of major organs in the management of nutrients and detoxification of the liver and kidneys (Ioniţă-Mîndrican et al., 2022). This finding can be attributed to the dietary fiber property that largely decreases levels of uremic toxins in the blood, and inhibits uremic toxin-producing bacteria’s growth, reducing toxins’ permeability into the blood. Urea and uremic toxins are associated with the kidneys losing the ability to effectively and efficiently filter waste out of the blood, leading to complications, including heart disease and even neurological episodes like seizures (Su et al., 2022).

Given what is known about the relationship between diet and brain health, this exploratory study sought to specifically examine the hypothesis that brain health would be associated with a healthy diet in healthy adults. Specifically, we predicted that gray matter volume would be correlated with total dietary calorie density and dietary fiber in a convenience sample of 190 participants from the Aging Brain Cohort (ABC@USC) study. Furthermore, we predicted that brain areas related to cognition and implicated in chronic diseases including dementia and Alzheimer’s disease, would show particularly strong correlations.

## Methods

This study was a cross-sectional design that used self-reported surveys such as the National Health Interview Survey Supplement 2015 Survey (2015 NHIS Questionnaire - Sample Adult Diet and Nutrition, 2016), a 31-question survey administered every 5 years covering topics such as diet and nutrition. Total and regional gray matter volumes were derived from magnetic resonance imaging (MRI). Diet fiber concentration and diet calorie density of each reported food were quantified using the NHIS 2015 questionnaire. Ethical approval for this study was obtained from the relevant institutional review board. All methods were performed in accordance with the relevant guidelines and regulations, ensuring adherence to ethical standards for research involving human subjects (von Elm et al., 2007). Informed consent was attained from all participants prior to participation in the study.

### Participants

Data from the NHIS 2015 Dietary Questionnaire was drawn from the University of South Carolina’s Aging Brain Cohort Study Repository a multimodal lifespan database for studying the relationship between the brain, cognition, genetics, and behavior in healthy aging. As part of this study, T1-weighted MRI images were collected, and participants also completed a dietary questionnaire. From the duration of September to 2019-December 2023, participants in the study underwent testing, filled out health-related forms and cognitive tests, and underwent an MRI scan using the University of South Carolina T1-weighted structural MRI scanner. The University of South Carolina IRB approved this procedure. The 190 participants were between 20 and 79 years old (Mean age = 48.284, 49 males, 141 females). To remain in suitable conditions for testing, participants had to stay in a supine position for nearly an hour, their maximum girth being lower than 60 inches, and maximum weight under 400 pounds. Notably, participants did not have existing medical conditions including, but not limited to: severe illnesses like cancer, untreated and unmanaged psychological conditions like schizophrenia, and no current or past fatiguing illnesses. In this study, the amount of participants was determined by the amount of data available for analysis as all available data was utilized. This dataset specified the sex, age, socioeconomic status, and race of each individual as fixed variables.

### Scanning Protocol

All participants underwent the same magnetic resonance imaging (MRI) scanning protocol at the University of South Carolina, McCausland Center for Brain Imaging on a Siemens Trio 3T scanner with a 20-channel head coil. T1-weighted images were used for volumetric analyses and were acquired using the following parameters: T1-weighted imaging (MP-RAGE) sequence with 1 mm isotropic voxels, 256×256 matrix size, 9° flip angle, and 92-slice sequence with repetition time = 2250 ms, inversion time = 925 ms, and echo time = 4.11 ms.

### Gray Matter Volume

To extract regional gray matter volumes, the Computational Anatomy Toolbox (CAT12) was employed with default settings. CAT12 for voxel-based morphometry (VBM) employs a fully automated workflow, a software tool that quantifies brain structure from MRI scans (Gaser et al., 2024). It parses the brain into different parts, adjusts the images to a standard brain atlas, and checks for errors. It then produces the size and thickness of various brain regions. Using voxel-based morphometry (VBM), the gray matter volume of specific brain regions was used to find the total distribution of gray matter throughout the cortex. This process starts with bias correction to minimize MRI artifacts and then segments the brain into gray matter (GM), white matter (WM), and cerebrospinal fluid (CSF) using tissue classification. The images are spatially normalized to an MNI template, corrected for local brain size, and modulated to preserve volume information. Finally, the processed data undergo statistical analysis to detect structural differences across groups. CAT12 produced the total gray matter volume of the entire subject, but also the gray matter volume of specific brain regions. The proportion of total gray matter in each area of the brain ($\frac{\mathrm{Regional GMV}}{\mathrm{Total brain GMV}}$) was found. The default settings used for this analysis include preprocessing steps, such as spatial registration to the reference brain, tissue segmentation, and correction of the intensity of non-uniformities. For further understanding of default settings, VBM, and CAT12, the following resource was utilized (Gaser et al., 2024).

### Fiber Concentration and Calorie-Density:

The concentration of fiber was calculated through a proportion of grams of fiber in food out of the total grams of food per serving using the official United States Department of Agriculture database (Food Surveys Research Group : USDA ARS, n.d.). If certain items were not found on the USDA site, the University of Rochester Medical Center Adult and Children’s Health Encyclopedia was utilized for additional information about fiber concentration within foods (Nutrition Facts - Current, n.d.). These same sources provided ample data on the calorie density of foods, which was calculated by taking the total calories per serving of each food divided by the grams per serving of each food. However, when cereals were evaluated, the SmartLabel (for Kellogg’s products) and box labels were utilized to estimate the most accurate fiber concentration and calorie density of each item.

However, in the question “How often did you eat CHOCOLATE, or any other types of CANDY? Do NOT include SUGAR-FREE CANDY” on the NHIS 2015, the 20 most popular chocolates and candies by market share, sales volume, and keyword traffic, were chosen and fiber concentration and calorie-density of the foods were averaged (Afzal, 2023). However, for questions of similar nature as “How often did you eat WHOLE GRAIN BREAD including toast, rolls and in sandwiches? Whole grain bread includes whole wheat, rye, oatmeal and pumpernickel”, the USDA fiber concentration and calorie-density values of whole wheat, rye, oatmeal, and pumpernickel where averaged to input single fiber-concentration and calorie-density for whole grain bread. A similar procedure was followed for questions with the same format, such as “How often did you eat COOKIES, CAKE, PIE, or BROWNIES?” question. In addition, for the NHIS 2015 question “How often did you eat POPCORN?”, three representative classic-type popcorn brands (Act ii, Pop Secret, Orville Redenbacher) were averaged to output average fiber concentration and calorie density for the popcorn variable. Finally, for the question “How often did you drink COFFEE or tea that had sugar or honey added to it? Include coffee and tea you sweetened yourself and presweetened tea and coffee drinks such as Arizona Iced Tea and Frappuccino”, the beverages that were averaged included a Starbucks Coffee Frappuccino with whole milk, Arizona Lemon Iced Tea, a Starbucks Medium Pike Place Roast, and a Starbucks Earl Gray Tea to find the fiber concentration and calorie-density.

In the study, participants not only had the option to input whether or not they consumed a specific food but also the frequency with which they consumed this food. Possible responses to this question were as such: Never (0 times per month), 1-time last month, 2-3 times last month, 1 time per week (Approximately 4 times per month), 2 times per week (Approximately 8 times per month), 3-4 times per week (Approximately 14 times per month), 5-6 times per week (Approximately 22 times per month), 1 time per day (Approximately 30 times per month), and 2 or more times a day (Approximately 60 times per month). These responses were given this number scale by increasing frequency per month receiving higher values on a scale from 1-9: Never (1), 1-time last month (2), 2-3 times last month (3), 1 time per week (4), 2 times per week (5), 3-4 times per week (6), 5-6 times per week (7), 1 time per day (8), and 2 or more times a day (9). Table 1 summarizes the demographic details of the 190-subject dataset utilized in this study.

### Statistical Methods:

To properly deduce correlations among fiber concentration and calorie density, an index must account for the frequency and each type of food consumed. As a result, an index was created and is defined as such: The 31-question survey filled out by participants covers 31 food items/groups. Each group calculated a fiber concentration and a calorie density that is representative of that question’s food items. Using the frequency of consumption inputted by the users for a question, this will be multiplied by the established fiber concentration (for fiber index) and established calorie-density (for calorie-density index). This operation will be completed for all 31 questions. Then, the 31 fiber indexes will be independently summed as well as the 31 calorie-density indexes to create a distinct fiber-concentration index (total fiber) and distinct calorie-density index (total calorie density) for the entire user-consumed diet. Using these total index variables, we can accurately compare the fiber and calorie density of a single participant’s diet using 1 variable, creating efficient comparisons between gray matter volume proportions.

Following the data sorting, we examined correlations between total calorie density total fiber concentration, and regional gray matter volume using JASP (Love et al., 2019). Specifically, spearman’s R-values and associated p-values were calculated to assess the statistical significance of the observed correlations between fiber-concentration indexes, calorie-density indexes, and gray matter volume proportions in participants. Given that the dietary index data (fiber and calorie density) are ordinal, based on frequency inputs, and may not follow a normal distribution, Spearman’s method provides a more accurate correlation measure compared to parametric tests like Pearson’s correlation. Furthermore, Spearman’s correlation is less affected by outliers, which is advantageous when working with diet-related data that can have extreme or skewed values.

## Results:

Complete data on demographic factors including age, sex, and race were available for all 190 analyzed subjects in the dataset. However, socioeconomic status was only available for 158 of these participants. Based on the non-normal distribution of some of the variables, we chose to use a series of Spearman’s rank-order correlation coefficient tests to assess the relationships between dietary fiber concentration, calorie density, and gray matter volume across various brain regions. The results in Table 2 reveal several noteworthy correlations between higher fiber consumption and consumption of calorie-dense foods and increased gray matter, particularly in areas involved in executive function and emotional processing.

### Correlations Between Variables of Interest

Spearman’s correlation test reveals results in Table 2 indicating a significant positive correlation between age and total dietary fiber consumed, indicating that elderly people in our study were associated with the consumption of high-fiber foods. The table reveals a significant negative correlation between the Race index and fiber consumed, indicating that patients of Caucasian descent (decreased race index of 1) likely had an association with higher fiber-based diets. Furthermore, Table 2 also reveals a significant negative correlation between race and total calorie-density per diet. This reveals that in our participant study, people of Caucasian/White descent were typically associated with a diet of increased calorie density, as a lower race index indicated higher calorie-dense consumption.

### Correlations Between Brain Volume and Dietary Metrics

**Figure 1.**
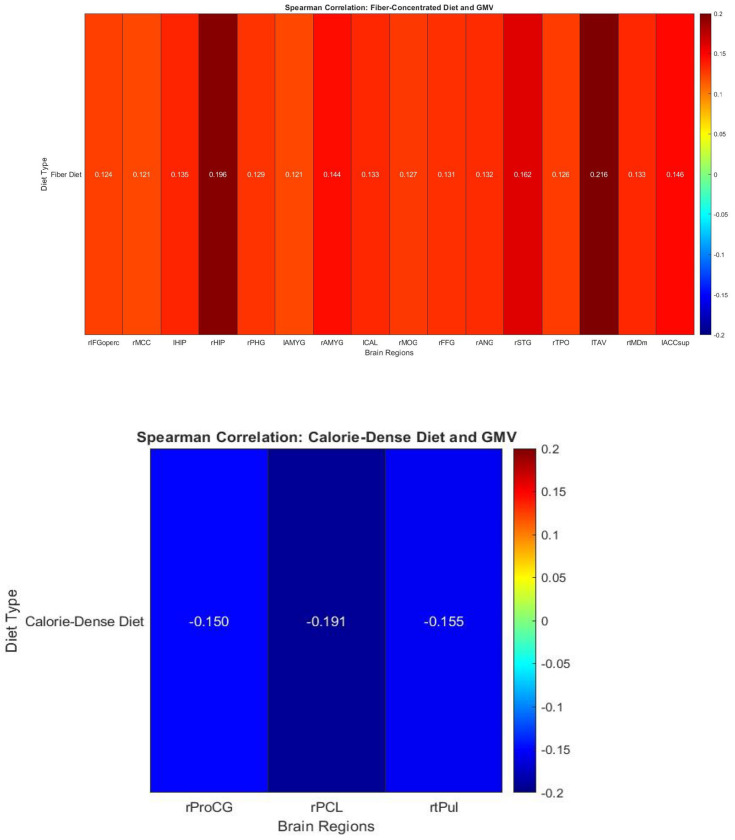
The results of the Spearman Correlation test run on the relations between calorie-dense diets & GMV as well as fiber-concentrated diets and GMV. Figures were generated as heatmaps using the MATLAB plot generator. Only results that yielded significance are visually present in the heatmap.

A second series of one-tailed Spearman’s correlation coefficient tests revealed significant correlations between a fiber-concentrated diet and gray matter concentration. The test controlled for the race of the participant and their age at the time of the test, as these two variables revealed significant correlations with the fiber concentration of a diet. Due to existing literature suggesting the cognitive benefits of fiber concentration, the alternative hypothesis was that the factors of fiber concentration in diet and GMV were correlated positively. Figure 1 shows a comparison representation of these results in heatmap format. The following areas yielded results including: the right Interior Frontal Gyrus (pars opercularis), rIFGoperc, **(r (190) = 0.124, p = 0.046);** right Middle Cingulate Cortex, rMCC, **(r (190) = 0.121, p = 0.05);** left Hippocampus, lHIP, **(r (190) = 0.135, p = 0.032);** right Hippocampus, rHIP, **(r (190) = 0.196, p = 0.003);** right Parahippocampal Gyrus, rPHG, **(r (190) = 0.129, p = 0.039);** left Amygdala, lAMYG, **(r (190) = 0.121, p = 0.05);** right Amygdala, rAMYG, **(r (190) = 0.144, p = 0.025);** left Calcarine Cortex, lCAL, **(r (190) = 0.133, p = 0.035);** right Middle Occipital Gyrus, rMOG, **(r (190) = 0.127, p = 0.041);** right Fusiform Gyrus, rFFG, **(r (190) = 0.131, p = 0.037);** right Angular Gyrus, rANG, **(r (190) = 0.132, p = 0.036);** right Superior Temporal Gyrus, rSTG, **(r (190) = 0.162, p = 0.013);** right Temporo-Parietal Occipital Junction (superior), rTPOsup, **(r (190) = 0.126, p = 0.043);** left Temporal Anterior Ventral area, lTAV, **(r (190) = 0.216, p = 0.001);** right Mediodorsal Thalamus, medial part, rtMDm, **(r (190) = 0.133, p = 0.035);** Intra-Anterior Cingulate Cortex, superior part, IACCsup, **(r (190) = 0.146, p = 0.023).**

**Figure 2.**
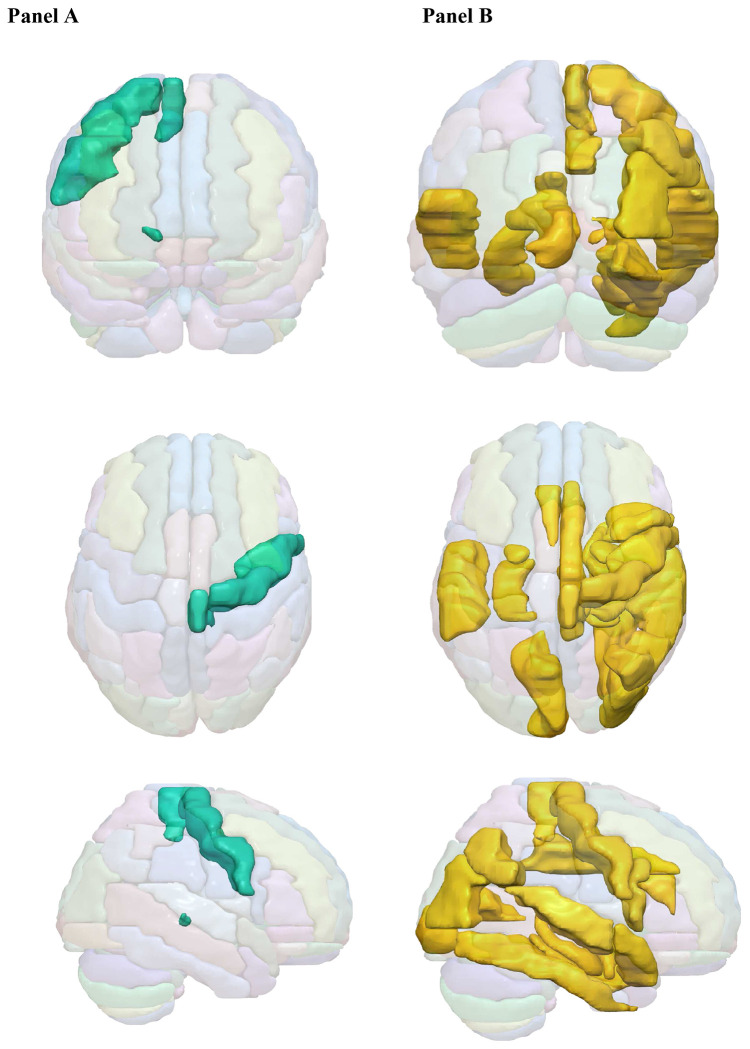
Graphical representation of brain regions related to relevant dietary variables of interest (calorie density = Panel A) and (dietary fiber = Panel B). Brain areas in which GMV was associated with calorie density included the following: rProCG, rPCL, and rtPul. Brain areas in which GMV was associated with fiber density included the following: rIFGoperc, rMCC, lHIP, rHIP, rPHG, lAMYG, rAMYG, lCAL, rMOG, rFFG, rANG, rSTG, rTPOsup, lTAV, rtMDm, and IACCsup.

A third series of Spearman’s correlation coefficient tests revealed other significant correlations between a calorie-dense diet and gray matter concentration. The race of participants was controlled for in the study, due to race and calorie density of diet being significantly correlated. This test was run as a two-tailed correlation test, with the alternative hypothesis being that calorie-density of diet and GMV were correlated. Results were revealed in the right Precentral Gyrus, rProCG, **(r (190) = −0.15, p = 0.039);** right Paracentral Lobule, rPCL, **(r (190) = −0.191, p = 0.008);** right Thalamic Pulvinar, rtPul, **(r (190) = −0.155, p = 0.033).** Figure 2 shows a visuospatial representation of areas of the brain affected by fiber-concentrated diets or calorie-dense diets.

## Discussion:

Our analysis suggests that there is a significant association between highly fiber-concentrated diets and GMV as well as calorie-density diet and GMV, with a high fiber-concentrated diet revealing positive correlations with GMV in localized brain areas and a highly calorie-dense diet revealing negative correlations with GMV in localized areas. The decay of GMV has been historically linked to a decreased risk of cognitive decline through neurodegenerative disorders. The current study used a cohort of subjects between 20 and 79 years of age; the study investigated the relationship between diets with variable fiber concentrations and calorie densities and the concentration of gray matter throughout various sections of the brain, using the NHIS 2015 Dietary Questionnaire to assemble a realistic indicator of the calorie density and fiber concentration of a subject’s diet. Based on prior empirical evidence, we predicted that a higher total fiber would provide valuable information for understanding gray matter decay in an aging subject, even after controlling for demographic variability in race, age, and socioeconomic status. However, due to a lack of adequate available data on the impact of calorie density on gray matter decay, we did not have enough prior empirical data to conclude calorie-dense diets and their association with the concentration of gray matter throughout the brain. Contrary to our expectations, high fiber concentration was not associated with higher gray matter throughout the brain but rather in a small set of brain areas including rIFGoperc, rMCC, lHIP, rHIP, rPHG, lAMYG, rAMYG, lCAL, rMOG, rFFG, rANG, rSTG, rTPO, lTAV, rtMDm and IACCsup. Similarly, high-calorie density was associated with decreased concentration of gray matter at three locations: rProCG, rPCL, and rtPul, suggesting that in localized brain regions, increased consumption of calorie-dense foods can contribute to cognitive decay.

These results are novel for several different reasons. First, this study examines how calorie density, a previously ignored factor of food data, affects structural changes within the brain, like gray matter. Previous research has studied how caloric restriction to an extent can foster healthy brain aging but has ignored the factor of how calorie-dense diets can contribute towards unhealthy brain maturity. Furthermore, several studies focused on dietary fiber changes and their contribution towards cognitive performance or brain atrophy tend to neglect the analysis of these factors on specific brain regions. This study’s use of gray matter volume (GMV) as a marker allows for a detailed examination of how dietary fiber and calorie density affect particular regions of the brain, such as the prefrontal cortex and hippocampus. This level of specificity enhances our understanding of how dietary changes influence not just general brain health but also region-specific structural integrity. Overall, this study suggests a possible pathway for researchers with MRI data to understand localized areas of the brain largely affected by dietary changes.

### Dietary Altercations and Gray Matter Volume:

Prior research widely associates larger dietary fiber intake with cognitive benefits, including decreased incidence of depression, higher-order cognition, and healthy aging, although there is a lack of studies emphasizing structural brain changes due to fiber intake. For example, one study reported that high diet quality, characterized by high fiber intake, has emerged as a notable factor in decreasing the incidence of mental health illnesses and the odds of acquiring depression. Furthermore, dietary patterns with high fiber concentration are also hypothesized to contribute to healthier brain structure during aging and have been seen to contribute to white matter microstructural integrity in animal models. In children and adolescents, high fiber intake has been seen to contribute towards improvements in executive functioning, such as working memory, but also episodic memory. Dietary fiber is considered a hallmark of the Mediterranean diet in addition to high concentrations of Omega-3 fatty acids. Elderly individuals who have followed the Mediterranean diet plan have reported reduced cognitive decline and alleviated cognitive impairment (Berding et al., 2021). Furthermore, dietary fiber has historically been associated with higher Omega-3 fatty acids, with foods like lentils, split peas, and black beans being common foods with high Omega-3 concentrations, which also have high fiber (Cleveland Clinic, 2023).

Gray Matter Volume was selected as part of the research study due to literature suggesting that gray matter volume is not only linked as a biomarker for neurodegenerative disorders but also because gray matter volume serves as an indicator for cognition. Recent studies reveal that those of older ages tend to exhibit smaller gray matter volume in the cerebellum which coincides with decreased scores in semantic memory, language processing, and retrieving/generating words compared to a middle age group. This suggests that gray matter volume is associated with overall cognition, creating significance in understanding factors that decrease this concentration (Ramanoël et al., 2018).

Another study suggests that higher dietary fiber intake is associated with larger DSST scores, suggesting that fiber consumption can contribute to a decreased risk of dementia and higher-order cognitive function. This result also suggests that a higher fiber-based diet can be indicative of higher information processing speed, working memory, and attention span, further legitimizing that fiber-concentrated foods can contribute to better cognition (Prokopidis et al., 2022). Results from our study are consistent with that of prior studies in that a higher fiber-concentrated diet contributed to higher GMV in localized brain areas, as a higher GMV is typically associated with higher cognitive performance (Droppa et al., 2017). This was even true after accounting for demographic differences like age, race, and socioeconomic status, suggesting that a higher fiber concentration accounts for GMV variability in certain localized brain regions. We would also like to make the point that existing studies regarding fiber-based diets and cognition largely did not study structural differences in the brain like GMV or localized areas of the brain largely affected by these diets. As a result, our results are important as they demonstrate the ability to specifically locate areas of the brain affected by dietary changes, using the Cat12 toolbox to isolate total GMV in a subject brain as well as GMV in a specific localized area of the subject’s brain.

Although existing literature does not address the effects of calorie-dense foods on structural changes in the brain or cognition, past studies have widely identified increased consumption of calorie-dense foods to be linked to an increased risk of acquiring conditions like obesity or diabetes. Recent epidemiological studies have concluded that diabetes mellitus (DM) is a major risk factor for developing cognitive dysfunction. This is largely due to the brain and neural tissue’s reliance on glucose as a substrate for energy. Thus, the alterations in carbohydrate metabolism that occur during diabetogenesis can directly impact cerebral and executive functioning, cognition, and memory. Alterations in the glucose transporters, hyper- and hypometabolism of the brain, and hypoglycemic episodes that are likely to occur in patients with diabetes are linked to neuronal damage. In addition, cognitive damage can directly affect DM, since diabetes treatment is largely a self-care process (Sebastian et al., 2023). Another source further states that obesity is also associated with reduced cognitive function and neuronal plasticity, implying that obesity is a large contributor to decaying brain structure. However, due to access to existing proven literature, there have not been many conclusions on the exact associations between obesity and cognitive decay (Sui & Pasco, 2020).

Results from our study are consistent with these existing findings. We would like to note that previous literature focuses specifically on diabetes and obesity as leading factors of cognitive decline but neglects the impact of merely consuming calorie-dense foods without either disorder. In our current study, we found that GMV in localized areas was decreased when the subject consumed increased calorie-dense foods listed in the NHIS 2015 Dietary Questionnaire. We found that the right Thalamic Pulvinar (rtPul), right Precentral Gyrus (rProCG), and right Paracentral Lobule (rPCL) were negatively associated with calorie density, which aligns with existing literature that consumption of calorie-dense foods, contributing to a risk of obesity and diabetes, may lead to cognitive decay. Due to a large gap in research on the association between the consumption of calorie-dense foods and structural changes in the brain, this study yields meaningful results as the study demonstrates that the consumption of calorie-dense foods referred to in the NHIS 2015 Dietary Questionnaire contributes to decreased cognitive function and brain structural integrity during aging.

### Automated Methods for MRI and Dietary Data Processing

The analysis of gray matter volume (GMV) and dietary data in research can be greatly enhanced by implementing automated methods that streamline the data processing workflow and minimize the need for manual intervention. When the researchers utilized the subject results from the NHIS 2015 Dietary Questionnaire to understand the total calorie density or total fiber concentration of each subject’s diet, the researchers had to first utilize the USDA or University of Rochester Medical Center Health Encyclopedia nutrition site to find fiber/serving and serving size for each food or to find calories/serving and serving size to find calories/gram for the calorie density variable (5 minutes for 1 reviewer per food). In some scenarios, if the food had different nutrition facts per brand (For example: popcorn brands Act ii, Pop Secret, and Orville Redenbacher all have different nutrition facts), the reviewer had to average the most popular brands of that food type (Up to 15 minutes for 1 reviewer per food).

When analyzing cereal nutrition facts, the researchers largely could not rely on the USDA or University of Rochester Medical Center Health Encyclopedia database due to their lack of cereal nutrition labels and had to rely on company websites (Kellogg’s SmartLabel) or pictures of nutrition labels to find the necessary fiber information or calorie density information (10 minutes for 1 reviewer per food). To expedite this process, machine learning algorithms can be trained to interpret free-text responses, allowing for more dynamic input handling in surveys like the NHIS 2015. In addition, creating a centralized dietary database that pulls nutritional information directly from validated sources like the USDA and University of Rochester Medical Center Health Encyclopedia databases can eliminate manual lookups, thereby reducing the risk of errors. Automated scripts can be developed to calculate dietary metrics, such as fiber concentration and calorie density, based on the standardized dietary codes. This not only has the potential to enhance efficiency but also ensures consistency, as calculations are based on standardized criteria rather than manual estimation. Integration of these methods within a single data processing pipeline can allow other researchers to utilize these automated processes, broadening the scope of dietary and brain structure research across different cohorts.

### Limitations and Future Directions:

Our study has some limitations that future researchers who aim to replicate our methods must understand. First, past studies conducted on fiber-concentrated diets and their impact on cognition typically utilized a *N* ≥ 1000: significantly larger amount of participants than the available at the University of South Carolina Aging Brain Cohort. If we had a larger group of participants, one could hypothesize that we would attain different results than the trends we recorded. Studies looking to adopt a similar methodology as this study should ensure that they have adequate available MRI data to test their results. A clear method for future research would be to available MRI data from larger deidentified clinical databases like the ADNI Alzheimer’s database (Mueller et al., 2005) where participants also have dietary data through questionnaires like the NHIS 2015 Dietary Questionnaire. Thus, while we did find significant relationships between high calorie-dense and highly fiber-concentrated diets with high localized GMV, we could have found different results if dealt with a larger, more detailed database.

A limitation of this study also derives from the lack of a fixed value for fiber concentration or calorie density of certain foods. The NHIS 2015 Dietary Questionnaire has questions like “How often did you eat CHOCOLATE, or any other types of CANDY? Do NOT include SUGAR-FREE CANDY”; “How often did you eat WHOLE GRAIN BREAD including toast, rolls and in sandwiches? Whole grain bread includes whole wheat, rye, oatmeal and pumpernickel”; “How often did you eat COOKIES, CAKE, PIE, or BROWNIES?”; “How often did you eat POPCORN?”; “How often did you drink COFFEE or tea that had sugar or honey added to it? Include coffee and tea you sweetened yourself and presweetened tea and coffee drinks such as Arizona Iced Tea and Frappuccino”’

For the food/beverages of chocolate/candy, bread, cookies/cakes/pie/brownies, popcorn, and coffee/tea, there are no fixed fiber concentrations or calorie-density given by the USDA or University of Rochester Medical Center Health Encyclopedia, as each variety and brand of these foods/beverages have differing nutrition facts. As a result, the researchers decided to average together the most commonly consumed of each food type (refer to Methods). However, it is not feasible to average every type of food, thus, the average fiber concentration and average calorie density calculated for each food may not be entirely representative of the entire food class, but rather just representative of the types that were averaged. This could have skewed results in our data and assisted in indicating false results.

We also did not account for all confounding variables in our study during analysis. Although race, age, socioeconomic status, and demographic variables were accounted for during the analysis, other variables such as mental well-being, sleep duration, and physical activity levels were not collected nor controlled for during the analysis. Previous literature has stated that higher physical activity and exercises have been linked to greater GMV in the prefrontal cortex and the hippocampus, as the volume of the prefrontal cortex and hippocampus remain responsive to moderately intense exercises for 6-12 months (Erickson et al., 2014). In addition, one source states that the optimal sleep duration associated with peak GMV is 6.7-7 hours, indicating that subjects who sleep approximately this long could naturally have a larger GMV than their counterparts regardless of dietary changes (Kim et al., 2021). Lastly, mood disorders such as bipolar disorder, depression, and schizophrenia spectrum disorder have been largely associated with decreased levels of GMV in the hippocampus and other areas of the brain responsible for emotional processing (Brosch et al., 2022). Thus patients who had similar mood disorders may have also had naturally lower GMV than their counterparts due to mood disorders and independent of dietary changes. Thus, we were not able to control for a large amount of confounding variables beyond age, race, and socioeconomic status in our study, possibly resulting in skewed data. Larger studies, due to a higher incidence of data and the ability to control for several variables, would likely not have as large of an issue with controlling for confounding variables.

## Conclusion:

Our data provides novel evidence that fiber-concentrated diets and calorie-dense diets referred to from the NHIS 2015 Dietary Questionnaire may be linked to increased localized GMV in areas of the brain. Due to the slowing of the rate of decay of GMV, calorie-dense, fiber-concentrated diets may act as possible treatment methods to further slow the progression of neurodegenerative disorders like Alzheimer’s disease in elderly populations, making this study of much importance to those who study neurodegenerative diseases. These studies highlight the value of understanding macronutrients and food nutrient breakdown when understanding possible ways to encourage cognitive growth and improve healthy structural brain aging. It is suggested that future studies focus on how other macronutrients like carbohydrates, proteins, and fats contribute to GMV, or how specific foods from the NHIS 2015 Dietary Questionnaire may have an impact on cognition. Future studies should also aim to develop methods on more sophisticated nutrition trackers to more precisely record the total calorie density and fiber in the diet and the use of linear regression machine learning models to predict dietary effects on brain health and growth of neurodegenerative diseases.

## Figures and Tables

**Table 1:** Demographic variables of interest

Category	Measure	Valid	Miss	M±(SD)	Min	Max	Range
Demographics	Race	190	0	W (153), AA (27), A (9), NA (1)			
	Sex	190	0	N (M) = 49, N (F) = 141			
	Age	190	0	48.284±19.804	20	79	59
	SES	158	32	45468.42±35333.334	1250	175000	173750

Abbreviations: W, White; AA, African American; A, Asian; NA, Native American. Miss, Missing/unavailable data; M, Mean; SD, Standard Deviation; Min, Minimum; Max, Maximum; N (M), Number of male participants; N (F), Number of female participants; SES, socioeconomic status.

**Table 2:** Spearman’s rho for demographics and variables of interest

Variable	Value	Age	Sex	Race	SES
Total Dietary Fiber Concentration	n	190	190	190	158
	Spearman’s rho	**0.219**	0.102	**−0.158**	0.154
	p-value	**0.002**	0.162	**0.029**	0.054
Total Calorie Density in Diet	n	190	190	190	158
	Spearman’s rho	0.057	0.010	**−0.206**	0.067
	p-value	0.436	0.889	**0.004**	0.404

Note: Correlations between total dietary fiber concentration and total calorie density in diet and demographic factors of age, sex, race, & SES. Race was quantified as 1 (White), 2 (African American), and 3 (Other Race). Methods detail methods in which total dietary fiber and total calorie density were calculated. Significant correlations are bolded (*p* < 0. 05).

Abbreviations: SES, socioeconomic status; n, sample size.

## Data Availability

The datasets generated and/or analyzed during the current study are available from the corresponding author upon request.
